# Supplementary material for: L1 Cell Adhesion Molecule in Cancer, a Systematic Review on Domain-Specific Functions
Source: Int J Mol Sci. 2019 Aug 26;20(17):4180. doi: 10.3390/ijms20174180 (PMC6747497; doi:10.3390/ijms20174180)
Supplement: Supplementary file 1 [file ijms-20-04180-s001.pdf]

Supplementary material:

Table S1. Main results of studies.

Cancer Type	Cancer-associated Process Affected	Model System, Patient Material *,1	Main Findings	Intervention	L1CAM Expression Analysis	Antibodies	Reference
**L1CAM-FL (plasma membrane)**<br>**L1CAM-FL studies with unspecified domain-specific functions**							
Breast cancer<br>Other carcinomas	Migration<br>Adhesion<br>EMT<br>Gene Regulation	in vitro<br>Cancer cell lines:<br>Breast: MCF7, MDA-MB231;<br>Lung: A549, NCI-H69;<br>Colon: HCT116, SW480;<br>prostate: PC3, LNCaP;<br>cervical: HeLa;<br>Ovarian: A2780;<br>Renal: ACHN;<br>Fibrosarcoma: HT1080;<br>Osteosarcoma: Saos-2;<br>Leukaemia: K562, HL60, CCRF-CEM;<br>Melanoma: Malme-3M, SK-MEL-28; Glioblastoma: U251	• Tumor cells co-express L1CAM-FL and L1CAM-SV (lacking exon 2 and 27), with dominant expression of L1CAM-FL<br>• Expression of L1CAM mRNA and protein decreases with cell density in MCF7, and depends on presence of adherens junctions<br>• Overexpression of L1CAM-FL decreases junctional E-cadherin and promotes cell scattering and β-catenin/TCF signaling<br>• L1CAM-KD promotes cell-cell adhesion; migration is restored by both L1CAM and L1CAM-SV, but not by soluble L1CAM-ECD	Overexpression<br>L1CAM-FL<br>L1CAM-Δ26-31,Δ1177-1180 (L1CAM-SV)<br>L1CAM-ECD<br>KD (shRNA)	WB, RT-PCR, qPCR, FCM/FACS, IF	UJ127	[44]
Esophageal squamous cell carcinoma (ESCC)	Proliferation<br>Migration<br>Invasion Metastasis<br>Gene Regulation	in vitro<br>Cancer cell lines:<br>SHEEC, KYSE150, KYSE510, KYSE450	• L1CAM expression is significantly increased in ESCC compared to non-cancerous tissue<br>• L1CAM KD results in decreased cell proliferation, migration and invasiveness, whereas	Overexpression<br>L1CAM-wt<br>KD (siRNA, shRNA)	qPCR, IHC, IF, WB	2C2<br>AF277	[45]

		in vivo<br>Mouse, s.c.<br>Patient material	overexpression of L1CAM showed opposite effects<br>• L1CAM KD in vivo attenuates tumor growth and metastasis. L1CAM upregulates expression of ezrin via activating β1 integrin/ERK/AP1 signaling				
Extrahepatic cholangiocarcinoma (ECC)	Migration<br>Invasion	in vitro<br>Cancer cell lines:<br>EGI-1 (R12D-KRas), TFK-1(wt-KRas)	• L1CAM KD in EGI-cells decreases migration and invasion but not proliferation and survival.<br>• L1CAM KD in TKF-1 yields no viable cells.<br>• L1CAM KD in EGI-1 cells inhibits P-JNK, but not P-ERK or P-Akt<br>• Pharmacological JNK inhibition inhibits migration and invasion of EGI-1 cells	KD (shRNA)	FCM, WB, RT-PCR	Ab417<br>A10-A3	[46]
Gastric cancer (GC)	Proliferation Migration Invasion Chemoresistance Tumor Growth Metastasis	in vitro<br>Cancer cell lines:<br>MKN28, AGS, SGC7901, HGC27 BGC823<br>in vivo<br>Mouse, s.c., tail vein<br>Patient material<br>FFPE, mRNA	• Overexpression of L1CAM in tumor tissue correlates with poor prognosis<br>• Interference of L1CAM levels in vitro show correlation of L1CAM expression and proliferation, colony formation, migration, invasion and chemoresistance<br>• L1CAM KD in vivo reduces tumor growth and metastasis<br>• L1CAM-induced migration and invasion is inhibited by inhibition of PI3K/Akt signaling	Overexpression L1CAM-FL<br>KD (shRNA)	IHC, WB, qPCR	5G3	[49]

Gastric cancer (GC)	Proliferation Migration Invasion	in vitro<br>Cancer cell lines:<br>KATOIII, NUGC4, NUGC2, MKN1, SC-6-JCK, GCIY, MKN45, MKN74,MKN28, AZ521<br>in vivo<br>Patient samples:<br>mRNA	• L1CAM KD inhibits cell proliferation, migration, invasion in gastric cancer cell lines<br>• Levels of phosphorylated ERK were reduced upon L1CAM KD.<br>• Overexpression of L1CAM correlates with local tumor cell growth, distant metastasis and tumor stage<br>• High L1CAM expression is correlated with poor patient survival	KD (siRNA)	WB, qPCR	UJ127	[47]
Gallbladder carcinoma (GBC)	Cell growth<br>Migration<br>Invasion<br>Tumor growth Survival	in vitro<br>Cancer cell lines:<br>JCRB1033, SNU-308<br>in vivo<br>Mouse, s.c.	• L1CAM promotes GBC cell cycle progression, migration, invasion and adhesion in vitro<br>• L1CAM promotes activation of Akt and FAK, but not ERK in vitro<br>• Expression of L1CAM in vivo promotes tumor growth and decreases survival	Overexpression: L1CAM-FL<br>KD (shRNA)	WB, RT-PCR	UJ127.11	[48]
Glioblastoma (GBM)	Apoptosis Chemoresistance Gene transcription	in vitro<br>Cancer cell lines:<br>Glioblastoma:U373, A172, T98G;<br>Pancreatic: Panc<br>in vivo<br>Patient samples:<br>Primary GBM cells	• L1CAM expression is transcriptionally upregulated by TGF-β1 in GBM cell lines<br>• Expression of L1CAM and TGF-β1 increases upon differentiation of patient derived GBM cells<br>• L1CAM expression in GMB cell lines and differentiated patient cells increases chemoresistance by reducing apoptosis<br>• L1CAM expression suppresses transcription of caspase-8	Overexpression: L1CAM-FL<br>KD (siRNA, shRNA)	FCM, qPCR, WB	UJ127.11	[50]

Melanoma (MM)	Migration<br>Invasion<br>Gene transcription	in vitro<br><u>Cancer cell line</u>:<br>K1735-C11	• L1CAM promotes increased and sustained activation of the ERK pathway in the presence of serum or PDGF<br>• Activation of this induces expression of migration- and invasion-associated genes<br>• Induction of integrin $\alpha v\beta 3$ and rac-1 contributes directly to L1CAM-dependent haptotaxis, whereas induction of cathepsins-L and -B promotes matrix invasion	Overexpression:<br>L1CAM-FL	WB, FCM	Anti-L1 pAb<br>(L1-ECD)<br>UJ127	[51]
Melanoma (MM)	Gene transcription	in vitro<br><u>Cancer cell line</u>:<br>MeWo	• L1CAM KD causes a significant reduction in MAGE genes and Wnt target genes	KD (shRNA)	Transcriptome microarray	none	[52]
Neuroblastoma (NB)	Proliferation<br>Migration<br>Radioresistance	in vitro<br><u>Cancer cell lines</u>:<br>IMR-32 (MycN-amplified),<br>SK-N-SH<br>(non-MycN-amplified)	• L1CAM KD downregulates MycN and upregulates PTEN in IMR-32 cells<br>• L1CAM KD inhibits proliferation, migration and tumorsphere formation in IMR-32 cells<br>• Radiotherapy upregulates L1CAM and MycN expression	KD (siRNA)	WB	ab123990<br>(aa 1153-1182)	[53]
Non-small cell lung cancer (NSCLC)	Migration<br>Invasion<br>Tumorigenicity Metastasis	in vitro<br>Immortalized bronchial epithelial: HBE135;<br>NSCLC cell lines: NCI-H460, H460SM, NCI-H125, NCI-H1264<br>―――<br>in vivo,<br>Mouse, s.c., rat, endobronchial	• L1CAM overexpression correlates with poorer prognosis<br>• L1CAM KD decreases cell migration and invasiveness and ERK activation in vitro, but does not affect proliferation or activation of Src and FAK<br>• Reduced tumor formation and growth was observed in mic<br>• L1CAM KD inhibits tumor growth and metastasis in vivo	Overexpression:<br>L1CAM-FL<br>KD (shRNA)	WB, qPCR, IHC	UJ127.11	[54]

Ovarian carcinoma (OC)	Proliferation Migration Invasion Chemoresistance	in vitro<br>Cancer cell lines: HTB77, OVCAR3<br><br>Patient samples: Ascites, tumor lysates	• Membrane-bound L1CAM expression correlates with invasion and lymphogenic spread<br>• L1CAM reduces IL-1β expression and NF-κB activity in vitro	KD (siRNA)	WB, ELISA, qPCR, FCM	L1-11A<br>Unspecified: (sandwich ELISA EIA5074)	[34]
Ovarian carcinoma (OC)	Proliferation<br>Apoptosis<br>Chemoresistance<br>Adhesion<br>Invasion<br>Transendothelial migraton	in vitro<br>Cancer cell lines: IGROV1, OVCAR3<br><br>Immortilized Primary ovarian surface epithelial cell lines: (OSE): HIO-80, IOSE80, IOSE29<br><br>Endothelial cells: HMEC-1, HUVEC, HDLEC	• L1CAM is highly expressed in normal ovarian surface epithelium (OSE), and expression is partially lost in epithelial OC (EOC), where it is only maintained at invasive regions and tumor center, and in a highly aggressive subset<br>• L1CAM positive tumors have poor prognosis, indicating differential functions of L1CAM in normal vs. tumor cells<br>• L1CAM promotes serum-dependent ERK activation, cell cycle progression and proliferation in tumor cells but not in immortalized OSE cells<br>• Serum-dependent L1CAM-stimulated proliferation depends on crosstalk with FGFR, but not EGFR<br>• L1CAM inhibits drug-induced apoptosis in EOC cells and promotes apoptosis in nontransformed OSE cells<br>• L1 inhibits intercellular adhesion in EOC cells and stimulates cell-cell adhesion in non-transformed OSE cells	Overexpression<br>L1CAM-FL<br>KD (siRNA)<br>Antibody blocking	IF, IHC	pcytL1<br>CE7<br>2C2<br>L1-S<br>(polyclonal, ECD)	[55]

		<u>Patient samples:</u><br>FFPE of normal ovarian tissue, cystadenomas, primary metastastatic tumor tissue	• L1CAM promotes invasion and transendothelial migration in EOC cells, but does not affect these processes. L1CAM-induced invasion involves crosstalk with FGFR and ERK and PI3K activation				
Retinoblastoma (RB)	Proliferation<br>Chemoresistance<br>Tumor growth<br>Adhesion	in vitro<br><u>Cancer cell lines:</u><br>Y79, SNUOT-Rb1<br>———<br>in vivo<br>Mouse, injection in vitreous cavity<br>Patient samples	• L1CAM overexpression in SNUOT-Rb1 promotes cell-cell adhesion, and LICAM KD in Y79 inhibits cell-cell adhesion<br>• Expression levels of L1CAM, modulated as above, positively correlate in vitro with: 1) proliferation, cell cycle progression and activation of p38 MAPK and Akt and in vivo with tumor growth and chemoresistance<br>• Patient data show negative correlation between L1CAM expression and retinoblastoma tumor differentiation	Overexpression<br>LICAM-FL<br>KD (shRNA)	IHC, WB, RT-PCR	2C2	[56]
Pancreatic carcinoma (PC)	Chemoresistance<br>Apoptosis	in vitro<br><u>Cancer cell lines:</u> PT45-P1, PT45-P1res, Colo357 Panc1	• L1CAM expression is upregulated in chemoresistant PT45-P1res cells in a IL1β-manner, and is required for chemoresistance in all tested PC cell lines<br>• Chemoresistance depends on IL1β-induced iNOS activation	Overexpression<br>L1CAM-FL<br>KD (siRNA)	qPCR, IHC, WB, FCM	UJ127<br>L1-11A<br>pCytL1	[58]

		Patient samples: FFPE	and NOS secretion, and results in caspase 3/7 inhibition<br>• L1CAM cleavage is not required for inducing chemoresistance<br>• L1CAM expression positively correlates with tumor grade in PC patients				
Pancreatic carcinoma (PC)	Chemoresistance<br>Apoptosis	in vitro<br>Cancer cell lines: PT45-P1, PT45-P1res, Colo357, Panc1	• Chemoresistant PT45-P1res has elevated expression of L1CAM expression as compared to parental line, but cell lines do not differ in heterophilic ligands α5-, ß1-integrin and neuropilin-1<br>• Blocking α5-,but not ß1-integrin abolishes chemoresistance in P45-P1res by inhibiting iNOS expression, and abolishing L1CAM-dependent caspase 3/7 inhibition<br>• L1CAM-induced chemoresistance relies on an intact RGD motif	KD (siRNA)<br>Overexpression<br>L1CAM-wt<br>L1CAM-D556E	WB, FCM	UJ127	[59]
Pancreatic carcinoma (PC)	Proliferation<br>Invasion	in vitro<br>Cancer cell lines: Capan2, PANC1, AsPC1, BxPC3, SW1990, Patu8988	• L1CAM KD in PC cells inhibits proliferation, cell cycle entry, invasion, and ERK activation. and invasion, but increased the number of cells in G0/G1 phase<br>• L1CAM KD in pancreatic cancer cells does not affect apoptosis	KD (siRNA, shRNA)	WB, RT-PCR, IF	unclear	[60]
Pancreatic carcinoma (PC)	Angiogenesis<br>Migration, Tubulogenesis<br>Adhesion<br>Gene regulation<br>Tumor growth<br>Survival	in vitro<br>Cancer cell lines: Panc02<br>Endothelial cell line: immortilized luEC (mouse)	• L1CAM expression is enhanced in blood vessels of human pancreatic carcinomas and other tumors<br>• Conditional endothelial KO of L1CAM or antibody blocking, promotes tumor vessel normalization, suppresses	Overexpression<br>L1CAM-FL<br>KD (siRNA)<br>KO (conditional KO; (Tie2-Cre; L1[floxed]<br>Antibody blocking	IHC, IF, WB, qPCR	S10-33<br>L-CI.5s (555;<br>clone 324<br>clone I4.2<br>clone UJ127<br>polyclonal<br>anti-Fc-	[57]

		in vivo<br>Mouse, conditional endothelial KO; intrapancreatic injection<br><br>Patient samples:<br>Tumor tissue, FFPE	tumor growth and promotes survival in vivo<br>• Overexpression of L1 in endothelial cells (ECs) promotes proliferation, migration, tubulogenesis, vascular permeability, and transcriptional reprogramming towards endothelial-to-mesenchymal transition (EMT) in vitro<br>• Inhibition of IL-6/JAK/STAT signaling prevents L1CAM-induced endothelial cell proliferation and migration			L1CAM<br>(blocking)	
T-cell lymphoma<br>Ovarian carcinoma (OC)	Migration<br>Invasion<br>Metastasis	in vitro<br>Cancer cell lines:<br>T-cell lymphoma: L-CI.5s (ESb;mouse) OC: SKOV3ip-lacZ<br><br>in vivo<br>Mouse, intradermal, tail vein	• L1CAM KD reduces migration and invasion in L-CI.5s and SKOV3ip but does not affect proliferation in vitro<br>• L1CAM KD does not affect growth of primary tumor of both cell lines in vivo, but promotes metastasis formation and growth.<br>• L1CAM KD decreases expression and activity of MMP-2 and MMP9	KD (shRNA)	IHC, IP,WB, FCM qPCR	L1-14.10<br>L1-9.3<br>L-CI.5s (555)	[61]
**L1CAM-FL studies with alternatively spliced isoforms**							
Breast cancer<br>Other carcinomas	Migration<br>Adhesion<br>EMT<br>Gene regulation	in vitro<br>Cancer cell lines:<br>Breast: breast MCF7, MDA-MB231<br>Lung A549, NCI-H69; Colon HCT116, SW480; Prostate PC3, LNCaP; Cervical HeLa; Ovarian A2780; renal ACHN<br>Fibrosarcoma HT1080,	• Tumor cells co-express L1CAM-FL and L1CAM and promotes cell scattering and β-catenin/TCF signaling<br>• L1CAM-KD promotes cell-cell adhesion; migration is restored by both L1CAM and L1CAM-SV, but not by soluble L1CAM-ECD -SV(lacking exon 2 and	Overexpression<br>L1CAM-FL<br>L1CAM-Δ26-31,Δ1177-1180<br>(L1CAM-SV)<br>L1CAM-ECD<br>KD (shRNA)	WB, RT-PCR, qPCR, FCM/FACS, IF	UJ127	[44]

		Osteosarcoma Saos-2. Leukemias: K562, HL60, CCRF-CEM. Melanoma: Malme-3M, SK-MEL-28. Glioblastoma: U251	27), with dominant expression of L1CAM-FL<br>• Expression of L1CAM mRNA and protein decreases with cell density in MCF7, and depends on presence of adherens junctions<br>• Overexpression of L1CAM-FL decreases junctional E-cadherin				
Ovarian carcinoma (OC)<br><br>Fibrosarcoma<br>Lymphoma<br>Colorectal cancer (CRC)	Metastasis<br>Gene regulation	in vitro<br><u>Cancer cell lines:</u><br>OvCar: SKOV3ip/lacZ<br>CRC: HCT-116,<br>T-Lymphoma: L-CI.5s (mouse)<br>Fibrosarcoma: HT1080/lacZ<br><br>in vivo<br>mouse, tail vein<br><br><u>Patient samples:</u><br>OC primary and metastatic tumor tissue (mRNA)	• L1CAM-FL and L1CAM-SV (lacking exons 2 and 27) mRNAs are both expressed in benign ovarian tumors and both increased during tumor progression<br>• Pro-metastatic growth factors upregulate L1CAM-FL at the expense of L1CAM-SV in OC and CRC cells<br>• Expression of L1CAM-FL, but not L1CAM-SV promotes metastasis of OC, CRC, fibrosarcoma and T-lymphoma cells in vivo<br>• L1CAM-FL, but not L1CAM-SV promotes expression and activation of MMP-2 and MMP-9<br>• L1CAM-FL is preferentially sorted in recycling and retrograde endosomes as compared to L1CAM-SV	Overexpression<br>L1CAM-FL<br>L1CAM-Δ26-31,Δ1177-1180 (L1CAM-SV)<br>Antibody-induced clustering	qPCR, WB	UJ 127.11 rabbit anti-human L1 (to L1CAM-ECD)	[62]

Ovarian carcinoma (OC)	Angiogenesis	in vitro<br>Cancer cell lines:<br>Endothelial cells (EC): Human ovarian cancer-derived ECs, vascular ECs, mouse lung derived ECs (luEC, lu2EC), embryo-derived ECs.<br>———<br>in vivo<br>Mouse models	• Alternative splicing of exon 25, encoding the L1CAM transmembrane domain, leads to expression of a novel soluble isoform in endothelial cells, named L1-ΔTM,,<br>• The splicing factor Nova2 is necessary and sufficient of alternative splicing of L1-ΔTM.<br>• L1-ΔTM has high angiogenic activity.<br>• L1-ΔTM-induced angiogenesis requires fibroblast growth factor receptor-1 signaling.<br>• Ovarian cancer vessels exhibit highly elevated co-expression of Nova2 and L1CAM as compared to vessels in normal tissue	Overexpression L1CAM wt L1CAMΔ1121-1143<br>KD (morpholino)<br>Recombinant L1-ΔTM protein	IF, IHC, WB, RT-PCR, qPCR	mAb 324	[11]
**L1CAM-FL studies with ECD-specific functions**							
Breast cancer	Adhesion	in vitro<br>Cancer cell lines:<br>MDA-MB231-Fra2 (high L1CAM)	• Breast cancer cells can interact with endothelial cells by homotypic L1CAM interactions.<br>• Breast cancer cells can interact with endothelial cells by heterotypic interactions of L1CAM in tumor cells with ALCAM, but not ICAM or integrin $\alpha5\beta1$ on endothelial cells.	Overexpression: L1CAM-FL<br>KD (shRNA)<br>Antibody blocking	WB, FCM	UJ127.11<br>L1-9.3	[63]
Breast cancer<br>Epidermoid carcinoma	Proliferation Tumor growth	in vitro<br>Cancer cell lines:<br>MDA-MB231<br>Epidermoid: A431	• Mint3 Induces L1CAM expression in mouse embryonic fibroblasts (MEFs) and tumor associated fibroblasts (CAFs) downstream of HIF activation<br>• interaction between Mint-induced L1CAM in fibroblasts	KD (shRNA)	qPCR, IHC, WB	NCAM-L1 Antibody (C-20)<br>ab123990	[64]

		in vivo<br>Mouse	and integrin α5β1 on tumor cells promotes integrin-dependent proliferation of tumor cells.<br>• L1CAM-integrin interaction is associated with ERK activation in tumor cells				
Colorectal cancer (CRC)	Proliferation Migration Metastasis	in vitro<br>Cancer cell lines:<br>CRC cell lines: Ls174T, HCT116<br><br>in vivo<br>Mouse, injection in spleen	• LCAM-wt expression in CRC cells promotes proliferation and scrape wound closure in vitro and metastasis in vivo<br>• Above effects are inhibited by H210Q mutation, but not a D598N mutation<br>• Mutation-specific gene expression signatures upon expression of H210Q and D598N mutants in CRC cells<br>• L1CAM-wt, but not H201Q mutant induces elevation of CD10 Elevation of CD10 depends on activation of NF-kB signaling via ezrin, which is inhibited by the H210Q mutation<br>• L1CAM-S542P does not localize to plasma membrane	Overexpression:<br>L1CAM-wt<br>L1CAM-H210Q<br>L1CAM-E309K<br>L1CAM-S542P<br>L1CAM-D598N	IHC, IF, WB	anti-L1-ICD	[65]
Melanoma (MM)	Adhesion Transendothelial migration	in vitro<br>Cancer cell lines:<br>WM239, M21	• L1CAM on melanoma cells is ligand for αvβ3 on endothelial cells and is required for transendothelial migration of melanoma cells	Overexpression:<br>L1CAM-FL<br>Peptide blocking:<br>Recombinant peptides:<br>L1CAM-Ig6 RGD mutant Ig6 RAD peptide<br>Antibody blocking	IF, WB	Anti L1-Ig1-3 (inhibition homotypic binding)<br>Anti L1-Ig4-6 (inhibits αvβ3 interaction)	[66]

Ovarian carcinoma (OC)	Apoptosis<br>Migration<br>Invasion	in vitro<br><u>Cancer cell lines:</u><br>OVMz<br>non-tumor: CHO (Chinese hamster)<br>———<br><u>Patient material:</u><br>ascites	• L1CAM is present in both exosomal and apoptotic vesicles from ovarian carcinoma cell lines and in ascites of ovarian carcinoma patients.<br>• Hypoxia and apoptotic stimuli increase apoptotic L1CAM-containing vesicle production ADAM10-dependent shedding of L1CAM<br>• Exosomal and apoptotic vesicles contain LCAM-FL and distinct cytosolic cleavage forms. .<br>• L1CAM-ECD purified from human patient ascites promotes ERK activation and transmigration in vitro<br>• L1CAM-ECD binds to α5β1, αvβ5, and αvβ3, but not to α6 integrin	No	WB, IF, ELISA, FCM	UJ127.11<br>Anti-pcytL1<br>mAb 324	[16]
Ovarian carcinoma (OC)	Adhesion	in vitro<br><u>Cancer cell lines:</u><br>MO68 SKOV3, OVM, M130, GG.<br>Primary mesothelial cells from OC patients<br>———<br><u>Patient samples:</u><br>ascites, tumor tissue	• L1CAM expression is in ovarian carcinoma cells, and low in mesothelial cells<br>• NRP-1 expression is high in mesothelial cells, and low in ovarian carcinoma cells.<br>• In contrast<br>• NRP-1 is trans-interaction partner of ligand for L1CAM-ECD binding, allowing interaction of tumor cells with mesothelium	Recombinant L1CAM-ECD-Fc fusion protein interactions<br>Antibody blocking	WB, ELISA, FCM, IF	L1-11A	[67]

Ovarian carcinoma (OC)<br><br>Fibrosarcoma<br>Lymphoma<br>Colorectal cancer (CRC)	Metastasis<br>Gene regulation	in vitro<br><u>Cancer cell lines:</u><br>Ovarian:SKOV3ip/lacZ<br>Colon: HCT-116,<br>T-Lymphoma: L-CI.5s (mouse)<br>Fibrosarcoma: HT1080/lacZ<br>———<br>in vivo<br>Mouse, tail vein<br>———<br><u>Patient samples:</u><br>OC primary and metastatic tumor tissue (mRNA)	• L1CAM-FL and L1CAM-SV (lacking exons 2 and 27) mRNAs are both expressed in benign ovarian tumors and both increased during tumor progression<br>• Pro-metastatic growth factors upregulate L1CAM-FL at the expense of L1CAM-SV in OC and CRC cells<br>• Expression of L1CAM-FL, but not L1CAM-SV promotes metastasis of OC, CRC, fibrosarcoma and T-lymphoma cells in vivo<br>• L1CAM-FL, but not L1CAM-SV promotes expression and activation of MMP-2 and MMP-9<br>• L1CAM-FL is preferentially sorted in recycling and retrograde endosomes as compared to L1CAM-SV	Overexpression<br>L1CAM-FL<br>L1CAM-Δ26-31,Δ1177-1180 (L1CAM-SV)<br>Antibody-induced clustering	qPCR, WB	UJ 127.11 rabbit anti-human L1 (to L1CAM-ECD)	[62]
Ovarian carcinoma (OC)	Angiogenesis	in vitro<br><u>Cancer cell lines:</u><br>ovarian cancer-derived endothelial cells (EC) vascular ECs, mouse lung derived ECs (luEC, lu2EC), embryo-derived ECs.	• Alternative splicing of exon 25, encoding the L1CAM transmembrane domain, leads to expression of a novel soluble isoform in endothelial cells, named L1-ΔTM<br>• The splicing factor Nova2 is necessary and sufficient of alternative splicing of L1-ΔTM.	Overexpression<br>L1CAM wt<br>L1CAMΔ1121-1143<br>KD (morpholino)<br>Recombinant L1-ΔTM protein	IF, IHC, WB, RT-PCR, qPCR	mAb 324	[11]

		in vivo<br>Mouse models	• L1-ΔTM has high angiogenic activity<br>• L1-ΔTM-induced angiogenesis requires fibroblast growth factor receptor-1 signaling<br>• Ovarian cancer vessels exhibit highly elevated co-expression of Nova2 and L1CAM as compared to vessels in normal tissue				
Pancreatic carcinoma (PC)	ChemoresistanceApoptosis	in vitro<br><u>Cancer cell lines:</u><br>PT45-P1, PT45-P1res, Colo357, Panc1	• Chemorestistant PT45-P1res has elevated expression of L1CAM expression as compared to parental line, but cell lines do not differ in heterophilic ligands α5-, ß1-integrin and neuropilin-1<br>• Blocking α5-,but not ß1-integrin abolishes chemoresistance in P45-P1res by inhibiting iNOS expression, and abolishing L1CAM-dependent caspase 3/7 inhibition<br>• L1CAM-induced chemoresistance relies on an intact RGD motif	Overexpression<br>L1CAM-wt<br>L1CAM-D556E<br>KD (siRNA)	WB, FCM	UJ127	[59]
Pancreatic carcinoma (PC)	Proliferation<br>Tumor growth	in vitro<br><u>Cancer cell lines:</u><br>PT45-P1*, Panc	• L1CAM-FL, but not L1CAM-ECD or L1CAM-CT are associated with cell proliferation in vitro or tumor growth in vivo.<br>• L1CAM-FL overexpression induces upregulation of IL-1β and constitutive NF-kB activation and L1CAM KD inhibits NF-kB activation.	Overexpression:<br>L1CAM-FL<br>L1CAM-ECD<br>L1CAM-CT<br>L1CAM-D556E<br>KD (siRNA)<br>Antibody blocking	IF, WB, ELISA, FCM, qPCR	L1-11A<br>L1-9.3<br>L1-35.9<br>pcytL1<br>mAb745H7	[23]

		in vivo<br>Mouse, s.c.	• LICAM-FL-mediated activation relies on IL-1β expression.<br>• LICAM-induced NF-kB activation relies on RGD domain and integrin/integrin-linked kinase signaling, but not on proteolytic cleavage of L1CAM				
Pancreatic ductal adenocarcinoma (PDAC)<br>Breast cancer	Migration<br>Invasion<br>EMT	in vitro<br>Cancer cell lines:<br>Panc, (L1CAM+), BxPc3 (L1CAM-), PT45-P1<br>Breast: MDA-MB231	• TGF-β1 induces EMT and L1CAM expression in MDA-MB231 and PDAC cell lines<br>• Upregulation of L1CAM is accompanied by increased expression of IL-1β and NF-kB in MDA-MB231<br>• Decreased IL-1β and NF-kB expression upon depletion of L1CAM, ezrin, β1-integrin or FAK<br>• Overexpression of L1CAM but not L1CAM-RGD mutant, results in increased phosphorylation of FAK and Src<br>• FAK and Src phosphorylation is also inhibited when various part of the integrin signaling pathways was knocked down	Overexpression<br>L1CAM-wt<br>L1CAM-D556E<br>L1CAM-ΔCT<br>KD (siRNA)	WB, qPCR	L1-11A<br>L1-9.3	[68]
Tumor of unknown origin	Nuclear signaling, cell migration, invasion and tumor growth	in vitro<br>Tumorogenic cell line:<br>HEK293	• L1CAM-D556E impairs L1CAM-wt-induced increase of cell-cell binding, cell migration, invasion, and tumor growth in vitro<br>• L1CAM-D556E does not impair L1CAM-wt induced ERK activation	Overexpression<br>L1CAM-wt<br>L1CAM-D556E<br>L1CAM-T1247A S1248A<br>Antibody blocking	IF, WB, FC, qPCR	L1-11A<br>pcytL1	[69]

		in vivo<br>Mouse s.c.	• Both L1CAM-D556E and L1CAM-T1247A, S1248A impair L1CAM-wt-induced gene transcription<br>• L1CAM-wt, but not L1CAM-D556E or L1CAM-T1247A, S1248A can transfer to the nucleus (presence of cleavage fragments not assessed)				
**L1CAM-FL-cytoplasmic domain**							
Colorectal cancer<br>(CRC)	Proliferation, Metastasis<br>Gene transcription	in vitro<br><u>Cancer cell lines</u>:<br>Ls174T (L1CAM-),<br>SW480, HCT116<br>in vivo<br>Mouse, injection in spleen	• Expression of L1CAM-FL, but not L1CAM-ΔCT or<br>• L1CAM-CT promotes proliferation at low serum in Ls174T cells in vitro<br>• Expression of L1CAM-FL, but not L1CAM-ΔCT or L1CAM-CT in Ls174T cells confers metastatic capacity in vivo<br>• Both ADAM10 and L1CAM are targets of β-catenin/TCF/signaling<br>• Co-expression of ADAM10 and L1CAM promotes L1CAM ectodomain shedding and promotes tumorigenesis<br>• The transcriptional profile of L1CAM-overexpressing cells resembles that of colon carcinoma tissue	Overexpression<br>L1CAM-FL<br>L1CAM-ΔCT<br>L1CAM-CT	IHC, IF, WB	polyclonal against FL<br>(both CT and ECT)	[26]
Colorectal cancer<br>(CRC)	Proliferation<br>Migration<br>Metastasis	in vitro<br><u>Cancer cell lines</u>:<br>Ls174T (L1CAM -),<br>SW620	• L1CAM promotes NF-kB signaling and downstream activation of proliferation and migration in vitro by interacting with, and enhancing phosphorylation of suppressor IkB	Overexpression<br>L1CAM-FL<br>L1CAM-ΔCT (Δ1180-1257)<br>L1CAM-ΔCT (Δ1176-1257)	IHC, IF, WB	7B5<br>8D9	[70]

		in vivo<br>Mouse, injection in spleen<br>———<br>Human material<br>FFPE CRC tumor sections	• L1CAM promotes metastasis in vivo by a NF-kB-dependent mechanism<br>• Y1151 in the L1CAM cytodomain is essential for NF-kB activation and downstream activation of proliferation, migration and metastasis, but not for interaction with IkB<br>• Y1151 is required for the interaction of L1CAM with ezrin, which forms a complex with IkB andL1CAM and ezrin near the plasma membrane in CRC cells<br>• L1CAM, ezrin and activated NF-kB are co-expressed at the invasive front in human CRC tissue	L1CAM-4A<br>(4x Ala mutations in 1149-1153)<br>L1CAM-Y1151A<br>KD (shRNA)			
Colorectal cancer (CRC)	Proliferation, migration, Metastasis<br>Gene regulation	in vitro<br><u>Cancer cell lines</u>:<br>Ls174T (L1CAM-), DLD-1,HCT116<br>———<br>in vivo<br>Mouse s.c.	• L1CAM enhances ezrin phosphorylation via ROCK which is required for L1CAM-ezrin co-localisation at the plasma membrane to enhance cell migration<br>• IGFBP-2 gene expression is elevated by L1CAM and ezrin through NF-kB mediated transactivation of the gene promoter<br>• Overexpression of IGFBP-2 in L1CAM-negative cells enhances cell proliferation, migration, tumorigenesis and metastasis similar to L1CAM overexpression and suppression of IGFBP-2 showed opposite effects	Conditional overexpression:<br>L1CAM wt L1CAM Y1151A	WB, IF,	Rabbit anti-L1	[71]
Ovarian carcinoma (OC) colorectal cancer (CRC) tumor of unknown origin	Migration<br>Invasion<br>Gene transcription	in vitro<br><u>Cancer cell lines</u>:<br>Ovarian:SKOV3ip<br>CRC: SW707<br><u>Tumorigenic cell line:</u> HEK293	• L1CAM-wt, but not L1CAMΔCT or L1CAM-T1247A, S1248A (L1CAM-TS) promotes migration and invasion in vitro in HEK293<br>• L1CAM-TS reduces tumor growth compared to L1CAM-wt in HEK293 and SW707<br>• L1CAM-TS mutation impairs L1CAM-induced ERK and Src activation	L1CAM-wt<br>L1CAM-ΔCT<br>L1CAM-S1248A<br>L1CAM-T1247A<br>KD (siRNA)<br>Antibody blocking	FCM, IP, WB	L1-11A (blocking)<br>L1-9.3 (blocking)<br>L1-14.10 (blocking)<br>L1-38.12	[27]

		in vivo<br>Mouse i.p.	• L1CAM-wt, but not L1CAM-TS increases transcription of pro-invasive cathepsin B, b3-integrin and of transcription factors HOX9 and AP2 and inhibits the tumor suppressor CRABPII in HEK293 in vivo<br>• L1CAM antibody blocking inhibits ovarian tumor growth in vivo				
Ovarian carcinoma (OC)<br>Fibrosarcoma<br>Lymphoma<br>Colorectal cancer (CRC)	Metastasis<br>Gene regulation	in vitro<br>Cancer cell lines:<br>Ovarian:SKOV3ip/lacZ<br>CRC: HCT-116,<br>T-Lymphoma: L-CI.5s (mouse)<br>Fibrosarcoma: HT1080/lacZ<br>in vivo<br>Mouse, tail vein<br>Patient samples:<br>OC primary and metastastic tumor tissue (mRNA)	• L1CAM-FL and L1CAM-FL (lacking exons 2 and 27) mRNAs are both expressed in benign ovarian tumors and both increased during tumor progression<br>• Pro-metastatic growth factors upregulate L1CAM-FL at the expense of L1CAM-SV in OC and CRC cells<br>• Expression of L1CAM-FL, but not L1CAM-SV promotes metastasis of OC, CRC, fibrosarcoma and T-lymphoma cells in vivo<br>• L1CAM-FL, but not L1CAM-SV promotes expression and activation of MMP-2 and MMP-9<br>• L1CAM-FL is preferentially sorted in recycling and retrograde endosomes as compared to L1CAM-SV	Overexpression<br>L1CAM-FL<br>L1CAM-Δ26-31 Δ1177-1180 (L1CAM-SV)<br>Antibody-induced clustering	qPCR, WB	UJ 127.11 rabbit anti-human L1 (to L1CAM-ECD)	[62]
Pancreatic carcinoma (PC)	Proliferation<br>Tumor growth	in vitro<br>Cancer cell lines:<br>PT45-P1*, Panc<br>in vivo<br>Mouse, s.c,	• L1CAM-FL, but not L1CAM-ECD or L1CAM-CT are associated with cell proliferation in vitro or tumor growth in vivo<br>• L1CAM-FL overexpression induces upregulation of IL-1β and constitutive NF-kB activation and L1CAM KD inhibits NF-kB activation<br>• LICAM-FL-mediated activation relies on IL-1β expression.<br>• LICAM-induced NF-kB activation relies on RGD domain and integrin/integrin-linked kinase signaling, but not on proteolytic cleavage of L1CAM	KD (siRNA)<br>Overexpression:<br>L1CAM-FL<br>L1CAM-ECD<br>L1CAM-CT<br>L1CAM-D556E<br>Antibody blocking	IF, WB, ELISA, FCM, qPCR	L1-11A<br>L1-9.3<br>L1-35.9<br>pcytL1<br>mAb745H7	[23]

PDAC and breast carcinomas	Migration<br>Invasion<br>EMT	in vitro<br>Cancer cell lines:<br>Panc, (L1CAM+), BxPc3 (L1CAM-), PT45-P1<br><br>Breast: MDA-MB231	• TGF-β1 induces EMT and L1CAM expression in MDA-MB231 and PDAC cell lines<br>• Upregulation of L1CAM is accompanied by increased expression of IL-1β and NF-kB in MDA-MB231<br>• Decreased IL-1β and NF-kB expression upon depletion of L1CAM, ezrin, β1-integrin or FAK.<br>• Overexpression of L1CAM but not L1CAM-RGD mutant, results in increased phosphorylation of FAK and Src<br>• FAK and Src phosphorylation is also inhibited when various part of the integrin signaling pathways was knocked down	KD (siRNA)<br>Overexpression<br>L1CAM-wt<br>L1CAM-D556E<br>L1CAM ΔCT	WB, qPCR	L1-11A<br>L1-9.3	[68]
Pancreatic carcinoma (PC)	ERK activation	in vitro<br>Cancer cell lines:<br>COLO357 (FG, SG subclones), Panc1,<br><br>Human material<br>FFPE PC tumor tissue	• Paper mostly concerns mapping of antibody binding sites<br>• L1CAM is expressed in moderately and poorly differentiated PDAC tumors, generally at tumor margin.<br>• Tac-L1CAM chimera dimerization promotes ERK activation , which is inhibited in N1251 mutants<br>• N1251 mutation does not affect RanBPM binding	Overexpression chimeric proteins<br>Tac-ECD/L1CAM-CT:<br>Tac/L1CAMwt<br>Tac/LICAM-N1251A<br>Tac/L1CAM-N1251D	IHC, ELISA, IP, WB, FCM	2C2<br>C20<br>UJ127<br>NCAML1<br>(5G3)	[72]
Tumor of unknown origin.	Migration<br>Invasion<br>Gene transcription<br>Tumor growth	in vitro<br>Tumorigenic cell line: HEK293<br><br>in vivo<br>Mouse s.c.	• L1CAM-D556E impairs L1CAM-wt-induced increase of cell-cell binding, cell migration, invasion, and tumor growth in vitro<br>• L1CAM-D556E does not impair L1CAM-wt induced ERK activation<br>• Both L1CAM-D556E and L1CAM-T1247A, S1248A impair L1CAM-wt-induced gene transcription<br>• L1CAM-wt, but not L1CAM-D556E or L1CAM-T1247A, S1248A can transfer to the nucleus (presence of cleavage fragments not assessed)	L1CAM-wt<br>L1CAM-D556E<br>L1CAM-T1247A S1248A<br>Antibody blocking	IF, WB, FCM, qPCR	L1-11A<br>pcytL1	[69]
**Intracellular L1CAM-Cytoplasmic domain (L1CAM-CT)**							

Colorectal cancer (CRC)	Proliferation<br>Metastasis<br>Gene transcription	in vitro<br><u>Cancer cell lines</u>:<br>Ls174T (L1CAM-),<br>SW480, HCT116<br>—<br>in vivo<br>Mouse, injection in spleen	• Expression of L1CAM-FL, but not L1CAM-ΔCT or<br>• L1CAM-CT promote proliferation at low serum in Ls174T cells in vitro<br>• Expression of L1CAM-FL, but not L1CAM-ΔCT or L1CAM-CT in Ls174T cells confers metastatic capacity in vivo<br>• Both ADAM10 and L1CAM are targets of beta-catenin/TCF/signaling<br>• Co-expression of ADAM10 and L1CAM promotes L1CAM ectodomain shedding and promotes tumorigenesis The transcriptional profile of L1CAM-overexpressing cells resembles that of colon carcinoma tissue	Overexpression<br>L1CAM-FL<br>L1CAM-ΔCT<br>L1CAM-CT	IHC, IF, WB	polyclonal against FL (both CT and ECT)	[26]
Glioblastoma (GB)	Radioresistance<br>Gene regulation	ex vivo<br>Mouse Xenografts from patient cells (see below)<br>—<br><u>Patient material:</u><br>GSCs and non-stem tumur cells (non-GSCs)	• L1CAM is highly expressed in a glioma subset with stem cell features and high radioresistance (Glioma stem cells; GSM)<br>• DNA damage induces L1CAM expression in GSCs<br>• L1CAM protects GSM from radiation damage through NBS1, which is required for activation of the checkpoint protein ATM kinase<br>• L1CAM-induced NBS1 relies on nuclear translocation of L1CAM-CT, which promotes Myc transcription and downstream expression of NBS1	Overexpression<br>L1CAM-CT<br>KD (shRNA)	IF, WB, qPCR	UJ127<br>C20 (SC-1508)	[79]
Ovarian carcinoma (OC)	Apoptosis<br>Migration<br>invasion	in vitro<br><u>Cancer cell lines</u>:<br>OVMz<br>non-tumor: CHO (Chinese hamster)	• L1CAM is present in both exosomal and apoptotic vesicles from ovarian carcinoma cell lines and in ascites of ovarian carcinoma patients.<br>• Hypoxia and apoptotic stimuli increase apoptotic L1CAM-containing vesicle production ADAM10-dependent shedding of L1CAM	No	WB, IF, ELISA, FCM	UJ127.11<br>Anti-pcytL1<br>mAb 324	[16]

		Patient material: ascites	• Exosomal and apoptotic vesicles contain LCAM-FL and distinct cytosolic cleavage forms<br>• L1CAM-ECD purified from human patient ascites promotes ERK activation and transmigration in vitro<br>• L1CAM-ECD binds to α5β1, αvβ5, and αvβ3, but not to α6 integrin				
Ovarian carcinoma (OC)<br>Pancreatic carcinoma (PC)	Gene transcription	in vitro<br>Cancer cell lines:<br>OVMz<br>non-tumor: CHO (Chinese hamster)<br>PC: PT45-PI<br>———<br>Tumorigenic cell line: HEK293	• Nuclear translocation of L1CAM in L1CAM-FL overexpressing cells depends on processing to L1CAM-CT by a metalloproteases and presenilin in HEK293 and OVMz cells<br>• Gene transcription regulation of cathepsin B (up) and CRABPII (down) in HEK293 and b3 integrin (up/OVMz) depends on L1CAM proteolysis by metalloproteases and presenilin<br>• L1-CAM dependent gene transcription of b3-integrin and CRABPII,is also induced by expression of L1CAM-CT in PT45-P1<br>• L1CAM-dependent ERK activation does not depend on metalloproteases and presenilin	Overexpression<br>L1CAM-FL<br>L1CAM-CT	FCM, WB, IF	L1-11A<br>mAb 745H7<br>Anti-pcytL1	[15]
Pancreatic carcinoma (PC)	Proliferation<br>Tumor growth	in vitro<br>Cancer cell lines:<br>PT45-P1*, Panc<br>———<br>in vivo<br>Mouse, s.c.	• L1CAM-FL, but not L1CAM-CT is associated with cell proliferation in vitro or tumor growth in vivo	KD (siRNA)<br>Overexpression:<br>L1CAM-FL, L1CAM-ECD,<br>L1CAM-CT<br>L1CAM-D556E<br>Antibody blocking	IF, WB, ELISA, FCM, qPCR	L1-11A<br>L1-9.3<br>L1-35.9<br>pcytL1<br>mAb745H7	[23]
**Extracellular L1CAM - Soluble ectodomain (ECD)**							
Breast cancer<br>Other carcinomas	Migration<br>Cell-cell adhesion<br>EMT<br>Gene regulation	in vitro<br>Cancer cell lines:<br>Breast: MCF7, MDA-MB231; Lung: A549, NCI-H69; Colon: HCT116, SW480; prostate: PC3, LNCaP; cervical: HeLa; Ovarian: A2780;	• Tumor cells co-express L1CAM-FL and L1CAM-SV(lacking exon 2 and 27), with dominant expression of L1CAM-FL<br>• Expression of L1CAM mRNA and protein decreases with cell density in MCF7, and depends on presence of adherens junctions	KD (shRNA)<br>Overexpression<br>L1CAM-FL<br>L1CAM-Δ26-31,Δ1177-1180<br>(L1CAM-SV)<br>L1CAM-ECD	WB, RT-PCR, qPCR, FCM/FACS, IF	UJ127	[44]

		Renal: ACHN;<br>Fibrosarcoma: HT1080;<br>Osteosarcoma: Saos-2;<br>Leukaemia: K562, HL60, CCRF-CEM;<br>Melanoma: Malme-3M, SK-MEL-28; Glioblastoma: U251	• Overexpression of L1CAM-FL decreases junctional E-cadherin and promotes cell scattering and b-catenin/TCF signaling<br>• L1CAM-KD promotes cell-cell adhesion; migration is restored by both L1CAM and L1CAM-SV, but not by soluble L1CAM-ECD				
Glioma	Migration	in vitro<br><u>Glioma cell lines:</u><br>9L/LacZ (rat), C6/LacZ (rat), U-87/LacZ,<br>Fibrosarcoma cell line QT6 (quail)<br><br><u>Patient material:</u><br>lysates, primary cells and frozen sections	• Fresh surgical sample cells co-express ADAM10 and L1CAM-FL, but ECD shedding is low/not detected<br>• Cultured cells from surgical samples and cell lines produce and shed L1CAM-ECD and L1CAM-ΔECD<br>• Exo-L1CAM-ECD and exo-L1CAM-ΔECD is found in exosomes, isolated from human U-86/LacZ, but not in exosomes from rat cell lines<br>• Glioma cell lines express L1-binding $\alpha v\beta 5$ integrin cell surface receptors<br>• Glioma cell migration is L1CAM KD or RGD-domain blocking antibodies	KD (anti-sense RNA)<br>Overexpression: L1CAM-FL<br>Antibody blocking	WB, RT-PCR, FCM, IF	sc-1508<br>UJ127<br>Lagenaur anti-L1<br>ASCS4 (blocking)<br>EZ1 (blocking)	[40]
Glioma<br>Breast cancer	Migration<br>Invasion	in vitro<br><u>Cancer cell lines:</u><br>T98G U-118 MG<br>Fibrosarcoma: QT6 (quail)<br>Breast: MDA-MB-231<br><br>in vivo<br>microinjection chick embryonic brain<br><br><u>Patient material:</u><br>Primary GBM cells and GBM lysates	• T98G cells shed extracellular L1CAM as L1CAM-ECD and as exo-FL<br>• L1CAM is co-expressed with ADAM10 in human glioma cells and correlates with L1CAM-ECD shedding<br>• L1CAM-ECD shedding occurs primarily at scrape wound edges in vitro<br>• L1CAM-ECD stimulates GBM cell migration in vitro and invasion in vivo<br>• LICAM-ECD promotes FAK activation, and is proposed to occur by interaction of L1CAM with integrins	KD (shRNA)<br>Overexpression: L1CAM-FL<br>L1CAM-ECD<br>peptide blocking: recombinant Ig6 RGD motif	IF, WB, RT-PCR	UJ127<br>NCAML1	[41]
Glioma	Migration<br>Proliferation	in vitro<br><u>Glioma cell lines:</u><br>T98G, U-118 MG	• L1CAM-ECD promotes proliferation and migration by an auto/paracrine mechanism<br>• Autocrine/paracrine activation of proliferation and migration is inhibited by	KD (shRNA)<br>Over expression<br>LICAM-ECD	WB	UJ127	[74]

			genetic or pharmacological inhibition of FGFR activity				
Glioblastoma (GB)	Proliferation, Cell cycle progression Migration	in vitro<br><u>Glioma cell lines:</u><br>T98G, U-118 MG	• Expression of L1CAM-FL or L1CAM-ECD promotes migration and cell cycle progression into S-phase<br>• Small molecule inhibitors of FGFR and FAK and RGD-peptide inhibition of $\alpha v\beta 3$ and $\alpha v\beta 5$ decrease L1-stimulated migration cell cycle progression	Overexpression L1CAM-FL L1CAM-ECD KD (shRNA)	None reported	None reported	[75]
Ovarian carcinoma (OC)	Apoptosis Migration invasion	in vitro<br><u>Cancer cell lines</u>:<br>OVMz<br>non-tumor: CHO (Chinese hamster)<br><br><u>Patient material:</u><br>ascites	• L1CAM is present in both exosomal and apoptotic vesicles from ovarian carcinoma cell lines and in ascites of ovarian carcinoma patients<br>• Hypoxia and apoptotic stimuli increase apoptotic L1CAM-containing vesicle production ADAM10-dependent shedding of L1CAM<br>• Exosomal and apoptotic vesicles contain LCAM-FL and distinct cytosolic cleavage forms<br>• L1CAM-ECD purified from human patient ascites promote ERK activation and transmigration in vitro<br>• L1CAM-ECD binds to $\alpha 5\beta 1$, $\alpha v\beta 5$, and $\alpha v\beta 3$, but not to $\alpha 6$ integrin	soluble L1CAM-ECD from ascites fluid	WB, IF, ELISA, FCM	UJ127.11 Anti-pcytL1 mAb 324	[16]
Ovarian carcinoma (OC)	Adhesion	in vitro<br><u>Cancer cell lines</u>:<br>MO68 SKOV3, OVM, M130, GG.<br>Primary mesothelial cells from OC patients<br><br><u>Patient material:</u><br>ascites, OC tumor tissue	• L1CAM expression is in ovarian carcinoma cells, and low in mesothelial cells<br>• NRP-1 expression is high in mesothelial cells, and low in ovarian carcinoma cells<br>• NRP-1 is trans-interaction partner of ligand for L1CAM-ECD binding, allowing interaction of tumor cells with mesothelium	Recombinant L1CAM-ECD-Fc fusion protein interactions Antibody blocking	WB, ELISA, FCM, IF	L1-11A	[67]

Ovarian carcinoma (OC)<br><br>Colorectal cancer (CRC)	Apoptosis<br>Chemoresistance<br>Gene regulation	in vitro<br><u>Cancer cell lines</u>:<br>OVMz M130<br>non-tumor: CHO (Chinese hamster)<br>CRC: SW707<br>——<br><u>Tumorigenic cell line</u>: HEK293<br>——<br><u>Patient material:</u><br>ascites, OC tumor tissue	• L1CAM expression protects against chemical stimulus- and hypoxia-induced apoptosis in HEK293 and CHO cells<br>• L1CAM expression upregulates ERK and FAK activation, and upon apoptotic stimuli, Bcl-2<br>• Purified L1CAM-ECD does activate FAK, but not protect against apoptosis in HEK293<br>• Long-term cisplatin treatment increases L1CAM expression in M130 and SW707 cells<br>• KD of L1CAM sensitizes cells for apoptosis	KD (siRNA)<br>Overexpression:<br>L1CAM-FL<br>soluble L1-CAM-ECD from ascites fluid	WB, FCM	UJ 127.11<br>pcytL1	[76]
Ovarian carcinoma (OC)	Apoptosis<br>Proliferation	in vitro<br><u>Cancer cell line:</u><br>SKOV3ip	• Serum and HGF promote shedding of L1CAM-ECD<br>• L1CAM-blocking antibody chCE7 synergize with tyrosine kinase inhibitor genestein to inhibit proliferation and induce apoptosis<br>• L1CAM-blocking antibody chCE7 synergize with tyrosine kinase inhibitor genestein to inhibit ERK, Src and Akt	Overexpression<br>L1CAM-ECD<br>Recombinant<br>L1CAM-ECD-Fc fusion protein<br>Antibody blocking	IP, WB, slot-blot	L1-11A<br><br>chCE7 (blocking)	[33]
Ovarian carcinoma (OC)	Angiogenesis	in vitro<br>Endothelial cells:<br>Bovine aortic endothelial (BEA)<br>OC cell line: SKOV3ip<br>——<br>in vivo<br>Chicken, chicken embryo chorioallantoic membrane (CAM)<br>angiogenesis assay	• RGD-dependent adhesion of SKOV3ip cells on L1Ig1-6 is blocked<br>• with mAb chCE7 but not with mAb UJ127.11.<br>• mAb chCE7 inhibits L1CAM-ECD-induced growth and matrigel invasion of BAE cells in vitro and tubulogenesis and angiogenesis in vivo<br>• sL1CAM-ECD binds to the $\alpha3$ integrin and induce activation of VEGFR-2 downstream of $\alpha v\beta3$ integrin in BEA cells	Overexpression<br>L1CAM-FL<br>L1CAM-ECD<br>L1CAM-ECD truncations (Ig1-4. Ig1-6, Ig1-6)<br>Recombinant<br>L1CAM-ECD peptides (ECD, Ig1-5, Ig1-6)<br>Antibody blocking	WB, slot-blot	chCE7 (blocking)<br>UJ127.11	[77]
Ovarian carcinoma (OC)	chemoresistance<br>Gene transcription	in vitro<br><u>Cancer cell line:</u><br>HTB77, OVCAR3<br>——<br><u>Patient material:</u><br>ascites, tumor lysates	• Soluble LICAM in patients correlates with increased chemoresistance and decreased survival<br>• L1CAM reduces IL-1β expression and NF-κB activity in vitro	KD (siRNA)	WB, ELISA, qPCR, FCM	L1-11A<br>Unspecified (sandwich ELISA EIA5074)	[34]

PDAC	Invasion/ Cancerous nerve invasion (CNI)	in vitro<br>Cancer cell line:<br>MiaPaCa2<br>KPC989 (mouse)<br>Immortalized Schwann cells:<br>SW10 (mouse)<br>Co-culture model<br><br>in vivo<br>KPC mouse, (Pdx-1-Cre/KrasG12D /p53R172H)<br><br>Patient material:<br>FFPE, normal tissue, PIN, PDAC	• Nerves and cancer cells express L1CAM at the perineural niche of invaded nerves in patients in vivo<br>• Schwann cells shed L1CAM-ECD in ADAM10-dependent manner<br>• Recombinant and Schwann cell-derived L1CAM-ECD is chemoattactant for PDAC cells, and induces invasion but not proliferation<br>• Schwann-cell L1CAM-ECD engages in homotypic interactions with PDAC L1CAM<br>• L1CAM-ECD causes ERK activation in PDAC in vitro<br>• L1CAM-ECD induces expression of MMP-2 and -9 through STAT3 activation<br>• CNI is blocked by a L1CAM-blocking antibody in vivo	KD (shRNA)<br>Overexpression recombinant L1CAM-ECD (mouse)<br>Antibody blocking	IP, WB, IF, IHC	L-CI.5s (555) (blocking)<br><br>anti-L1CAM clone 324	[78]
**Exosomal (Exo-FL, Exo-ΔCT, Exo-ΔECD, Exo-CT)**							
Glioma	Migration	in vitro<br>Glioma cell lines:<br>9L/LacZ (rat), C6/LacZ (rat), U-87/LacZ,<br>Fibrosarcoma: QT6 (quail)<br><br>Patient material:<br>lysates, primary cells and frozen sections	• Fresh surgical sample cells co-express ADAM10 and L1CAM-FL, but ECD shedding is low/not detected<br>• Cultured cells from surgical samples and cell lines produce and shed L1CAM-ECD and L1CAM-ΔECD<br>• Exo-L1CAM-ECD and exo-L1CAM-ΔECD is found in exosomes, isolated from human U-86/LacZ, but not in exosomes from rat cell lines<br>• Glioma cell lines express L1-binding $\alpha v\beta 5$ integrin cell surface receptors.<br>• Glioma cell migration is L1CAM KD or RGD-domain blocking antibodies	KD (anti-sense RNA)<br>Overexpression: L1CAM-FL<br>Antibody blocking	WB, RT-PCR, FCM, IF	sc-1508<br>UJ127<br>Lagenaur anti-L1<br>ASCS4 (blocking)<br>EZ1 (blocking)	[40]
Glioblastoma (GBM)<br>Breast cancer	Migration invasion	in vitro<br>Cancer cell line:<br>GBM: T98G, U-118 MG<br>Fibrosarcoma: QT6 (quail)<br>Breast: MDA-MB-231	• T98G cells shed extracellular L1CAM as L1CAM-ECD and as exo-FL<br>• L1CAM is co-expressed with ADAM10 in human glioma cells and correlates with L1CAM-ECD shedding.	KD (shRNA)<br><br>Overexpression: L1CAM-FL<br>L1CAM-ECD	IF, WB, RT-PCR	UJ127<br>NCAML1	[41]

		in vivo<br>microinjection chick embryonic brain<br><br><u>Patient material:</u><br>Primary GBM cells and GBM lysates	• L1CAM-ECD shedding occurs primarily at scrape wound edges in vitro<br>• L1CAM-ECD stimulates GBM cell migration in vitro and invasion in vivo<br>• LICAM-ECD promotes FAK activation, and is proposed to occur by interaction of L1CAM with integrins	peptide blocking:<br>recombinant Ig6<br>RGD motif			
Ovarian carcinoma (OC)	Apoptosis<br>Migration<br>invasion	in vitro<br><u>Cancer cell line:</u><br>OVMz<br>non-tumor: CHO (Chinese hamster)<br><br><u>Patient material:</u><br>Ascites	• L1CAM is present in both exosomal and apoptotic vesicles from ovarian carcinoma cell lines and in ascites of ovarian carcinoma patients<br>• Hypoxia and apoptotic stimuli increase apoptotic L1CAM-containing vesicle production ADAM10-dependent shedding of L1CAM<br>• Exosomal and apoptotic vesicles contain LCAM-FL and distinct cytosolic cleavage forms<br>• L1CAM-ECD purified from human patient ascites promote ERK activation and transmigration in vitro<br>• L1CAM-ECD binds to $\alpha5\beta1$, $\alpha v\beta5$, and $\alpha v\beta3$, but not to $\alpha6$ integrin	No	WB, IF, ELISA, FCM	UJ127.11<br>Anti-pcytL1<br>mAb 324	[16]

[*,1] Cell lines are human, and derived from tumor indicated in first column, unless specified otherwise,
CNI: cancerous nerve invasion, CRC: colorecal cancer, ECC: extrahepatic cholangiocarcinoma, EMT: epithelial-mesenchymal transition, ESCC: esophageal squamous cell carcinoma, GBM: Glioblastoma, GC: gastric cancer, MM: malignant melanoma, NSCLC: non-small cell lung cancer, OC: ovarian carcinoma, PC: pancreatic cancer, PDAC: pancreatic ductal adenocarcinoma, PDGF: platelet derived growth factor, PIN: pancreatic intraepithelial neoplasia, s.c.: subcuteneous, i.p.: intraperitoneally
ELISA: Enzyme-Linked Immuno Sorbent Assay, FACS: fluorescence activated cell sorting, FCM: flow cytometry, FFPE: formalin-fixed, paraffin-embedded IF: immunofluorescence, IHC: immunohistochemistry, IP: immunoprecipitation, KD: knockdown, qPCR: quantitative real time PCR, RT-PCR; reverse transcriptase PCR, WB: Western Blot.

Supplementary file S2: Search Strategy

S2.1 Search syntax PubMed

"Neoplasms"[Mesh] OR Cancer *[tiab] OR Malignanc *[tiab] OR Tumour *[tiab] OR Tumor*[tiab] OR Neoplasm [tiab] OR Neoplasia[tiab] OR Benign Neoplasms[tiab] OR Neoplasms, Benign[tiab] OR benign Neoplasm[tiab] OR Neoplasm, Benign[tiab]

AND

"Neural Cell Adhesion Molecule L1"[Mesh] OR L1 cell adhesion molecule [tiab] OR L1CAM[tiab] OR Neural Cell Adhesion Molecule L1[tiab] OR Neural Adhesion Molecule L1[tiab] OR Cell Adhesion Molecule L1[tiab] OR CD171[tiab] OR NILE glycoprotein[tiab] OR Glycoprotein, NILE[tiab] OR Nerve Growth Factor-Inducible Large External Glycoprotein[tiab] OR Nerve Growth Factor Inducible Large External Glycoprotein[tiab] OR CALL Protein[tiab] OR CamL1 Gene Product[tiab] OR NILE Protein[tiab]

S2.2 Search syntax Embase:

(Neoplasm * OR Carcinoma * OR Cancer * OR Tumor OR Tumour OR Tumors OR Tumours OR Neoplasia OR Malignanc * OR Benign Neoplasm *):ti,ab,kw.

AND

(Neural Cell Adhesion Molecule L1 OR L1 cell adhesion molecule OR L1CAM OR Neural Cell Adhesion Molecule L1 OR Neural Adhesion Molecule L1 OR Cell Adhesion Molecule L1 OR CD171 OR NILE glycoprotein OR Glycoprotein, NILE OR Nerve Growth Factor-Inducible Large External Glycoprotein OR Nerve Growth Factor Inducible Large External Glycoprotein OR CALL Protein OR CamL1 Gene Product OR NILE Protein):ti,ab,kw.
